# Deciphering the biology of *Cryptophyllachora eurasiatica* gen. et sp. nov., an often cryptic pathogen of an allergenic weed, *Ambrosia artemisiifolia*

**DOI:** 10.1038/s41598-018-29102-5

**Published:** 2018-07-17

**Authors:** Levente Kiss, Gábor M. Kovács, Károly Bóka, Gyula Bohár, Krisztina Varga Bohárné, Márk Z. Németh, Susumu Takamatsu, Hyeon-Dong Shin, Vera Hayova, Claudia Nischwitz, Marion K. Seier, Harry C. Evans, Paul F. Cannon, Gavin James Ash, Roger G. Shivas, Heinz Müller-Schärer

**Affiliations:** 1 University of Southern Queensland, Centre for Crop Health, Toowoomba, Qld 4350 Australia; 2 Plant Protection Institute, Centre for Agricultural Research, Hungarian Academy of Sciences (MTA-ATK), Budapest, H-1525 Hungary; 3 Eötvös Loránd University, Institute of Biology, Department of Plant Anatomy, Budapest, H-1117 Hungary; 4 Biovéd 2005 Ltd., Kemenestaródfa, H-9923 Hungary; 5 Mie University, Graduate School of Bioresources, Tsu, 514-8507 Japan; 6 Korea University, Division of Environmental Science and Ecological Engineering, Seoul, 02841 Korea; 7 National Academy of Sciences of Ukraine, M.G. Kholodny Institute of Botany, Kyiv, 01004 Ukraine; 8 Utah State University, Department of Biology, Logan, UT 84322 USA; 9 CABI Europe-UK, Egham, Surrey TW20 9TY United Kingdom; 10 Royal Botanic Gardens, Jodrell Laboratory, Mycology Section, Kew, TW9 3AB United Kingdom; 11 University of Fribourg, Department of Biology/Ecology & Evolution, Fribourg, CH-1700 Switzerland

## Abstract

A little known, unculturable ascomycete, referred to as *Phyllachora ambrosiae*, can destroy the inflorescences of *Ambrosia artemisiifolia*, an invasive agricultural weed and producer of highly allergenic pollen. The fungus often remains undetectable in ragweed populations. This work was conducted to understand its origin and pathogenesis, a prerequisite to consider its potential as a biocontrol agent. The methods used included light and transmission electron microscopy, nrDNA sequencing, phylogenetic analyses, artificial inoculations, and the examination of old herbarium and recent field specimens from Hungary, Korea, Ukraine and USA. The Eurasian and the North American specimens of this fungus were to represent two distinct, although closely related lineages that were only distantly related to other lineages within the *Ascomycota*. Consequently, we describe a new genus that includes *Cryptophyllachora eurasiatica* gen. et sp. nov. and *C. ambrosiae* comb. nov., respectively. The pathogenesis of *C. eurasiatica* was shown in *A. artemisiifolia*. No evidence was found for either seed-borne transmission or systemic infection. Two hypotheses were developed to explain the interaction between *C. eurasiatica* and *A. artemisiifolia*: (i) as yet undetected seed-borne transmissions and latent, systemic infections; or (ii) alternative hosts.

## Introduction

Common ragweed (*Ambrosia artemisiifolia*, Asteraceae) is an annual plant native to North America, known for its highly allergenic pollen as well as a major agricultural weed^1^. From the late 19^th^ century, its fruits (achenes) have spread around the world, including parts of Europe^2–4^, Asia^5,6^ and Australia^7^ as contaminants of agricultural goods but also through military activities. In many of these regions, *A. artemisiifolia* has become a serious invasive weed in agriculture, and has also triggered a dramatic increase in the number of people allergic to its pollen^8^. As a consequence, *A. artemisiifolia* has raised an awareness of the importance of alien weeds in Europe, unlike any other plant^1,8^.

In addition to chemical and mechanical methods of control, biological control has also been considered as a strategy to reduce populations of *A. artemisiifolia* in Europe^9,10^, China^11,12^ and Australia^13^. Biological control is a method of controlling pests with their natural enemies. Biotrophic fungal pathogens, especially rust fungi, have been used successfully as classical biological control agents (BCAs) against invasive weeds because of their host specificity and ability to cause devastating epidemics in introduced populations of the target plants^14^. Biotrophic fungal pathogens may not kill the infected host plant tissues quickly^15,16^, or even kill them at all^17,18^. However, some of these fungi can cause serious damage by preventing pollen production in male flowers^17,18^, increasing mortality of young trees^19^, reducing radial growth of older trees^20^, or through local extinction and fragmentation of plant populations^21^.

Amongst the few known fungal pathogens of *A. artemisiifolia*, a single candidate, the rust fungus *Puccinia xanthii*, was selected as a potential classical BCA in Europe^1^. Another potential candidate BCA is the little known ascomycete, *Phyllachora ambrosiae* that caused a serious epidemic in populations of *A. artemisiifolia* in Hungary in 1999, by destroying its inflorescences, stems and leaves^22^. Curiously, similar epidemics have not been observed in Hungary in subsequent years^8^, while serious infections have been developed in that period in *A. artemisiifolia* populations in Ukraine^23^. There is scant information about this apparently obligate biotrophic fungus, with the most recent comprehensive paper about its morphology and development in *A. artemisiifolia* tissues dating back more than 60 years, and being based on specimens collected in the USA^24^. So far, *P. ambrosiae* has only been recorded in the USA^24^, Hungary^22^ and Ukraine^23^. Two specimens deposited at Herbarium BPI (http://nt.ars-grin.gov/fungaldatabases/) substantiated its occurrence on *A. artemisiifolia* in Korea in 2003^25^, as well.

The absence of any detailed and up-to-date information about the identity, life cycle and pathogenesis of *P. ambrosiae* has hindered the assessment of its potential as a BCA^1^. This study was performed to (i) reveal the presence of *P. ambrosiae* in *A. artemisiifolia* populations established in Hungary, Korea, Ukraine and the USA based on long-term field surveys; (ii) determine the correct taxonomic placement of the fungus, based on molecular phylogenetic analyses of newly determined nrDNA loci; and (iii) reveal the pathogenesis and disease cycle in *A. artemisiifolia* based on light and transmission electron microscopy (TEM) studies of the infected plant tissues supplemented with inoculation experiments.

## Results

### Occurrence of *P. ambrosiae* in the field

**Figure 1:**
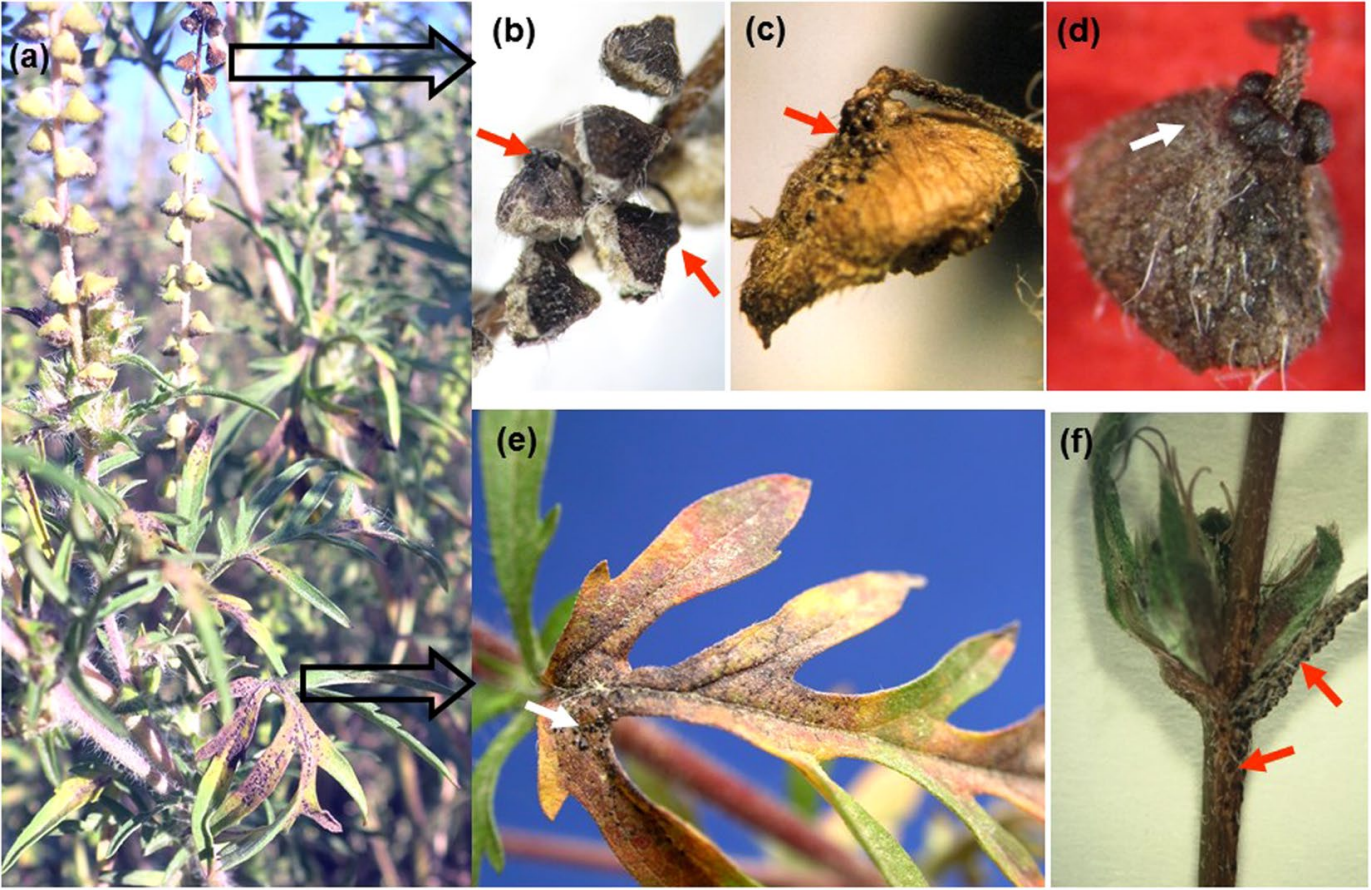
Symptoms of *Cryptophyllachora eurasiatica* infections of common ragweed (*Ambrosia artemisiifolia*) in the field. (**a**) Young plant exhibiting large, brownish lesions mainly on leaves. (**b**,**c**,**d**) Dry male inflorescences with mature perithecia (arrows). (**e**) Perithecia (arrows) on a leaf. (**f**) Perithecia (arrows) on a stem and around female inflorescences.

**Figure 2:**
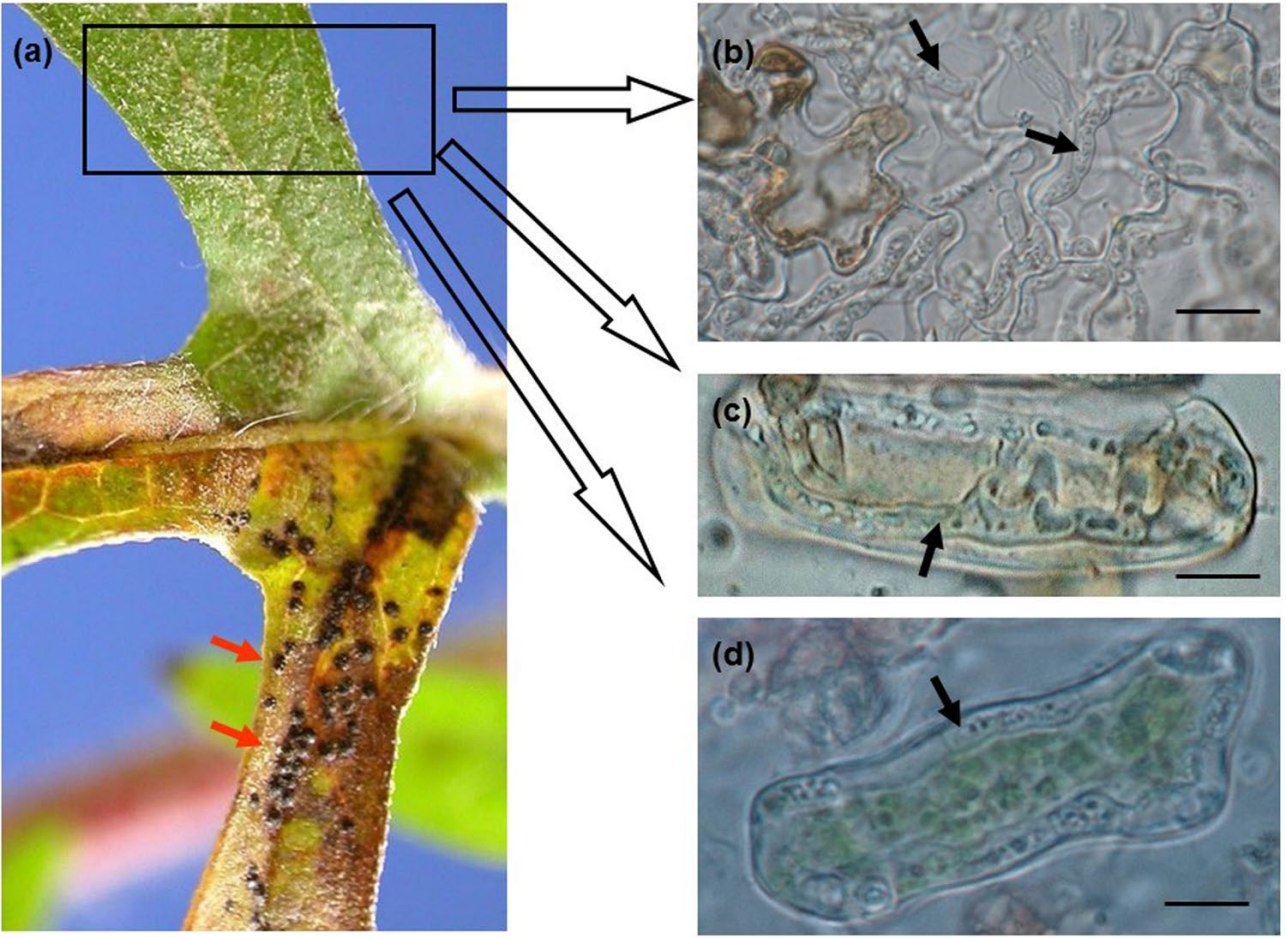
Inter- and intracellular hyphae of *Cryptophyllachora eurasiatica* in a common ragweed (*Ambrosia artemisiifolia*) leaf bearing mature perithecia. (**a**) Leaf segment with perithecia (arrows) on one side of the main vein, and no symptoms on the other side. (**b**) Hyphae (arrows) in an asymptomatic part of the leaf, decolorized in Carnoy’s solution. Bar = 20 µm. (**c**,**d**) Intracellular hyphae in mesophyll cells. Bar = 15 µm.

In Hungary, Korea and Ukraine, the occurrence of *A. artemisiifolia* plants exhibiting the typical symptoms of *P. ambrosiae* infections^22,23^ (Figs 1, 2a) was variable between locations, seasons and years. The fungus was found in some years, but not in others, at locations monitored for at least 5 years (Supplementary Table S1). In the USA, the fungus was first found in 2005 in Georgia in this study (Supplementary Table S2), but not in subsequent years in that region. Further specimens were collected in Florida in 2014, at two sites (Supplementary Table S2) that were surveyed only once in this study.

### Light microscopy

Perithecia and asci characteristic of *P. ambrosiae* were used to make tentative identifications of the fungus in field samples of *A. artemisiifolia* (Figs 1, 2). ITS sequences were used to confirm the identity of the pathogen. The hyphae in plant tissues were characteristically intracellular, sometimes intercellular, septate, 2–5 µm thick (Fig. 2b–d, Supplementary Fig. S1). ITS2 sequences determined in DNA samples extracted from leaf pieces containing such hyphae, but without perithecia or ascospores (as shown in Fig. 2a), always confirmed the presence of the fungus. Microscopic examination of tissue macerates was a simple and reliable diagnostic for the presence of intracellular hyphae of *P. ambrosiae* in parenchyma cells (Fig. 2c,d) even in the absence of perithecia. Intracellular hyphae were also easily detected in decolorized leaves (Supplementary Fig. S2). Semi-thin sections of infected leaves revealed the presence of intracellular hyphae in the upper and lower epidermis as well as both intra- and intercellular hyphae in the mesophyll (Supplementary Fig. S1).

### Study of old herbarium specimens

**Figure 3:**
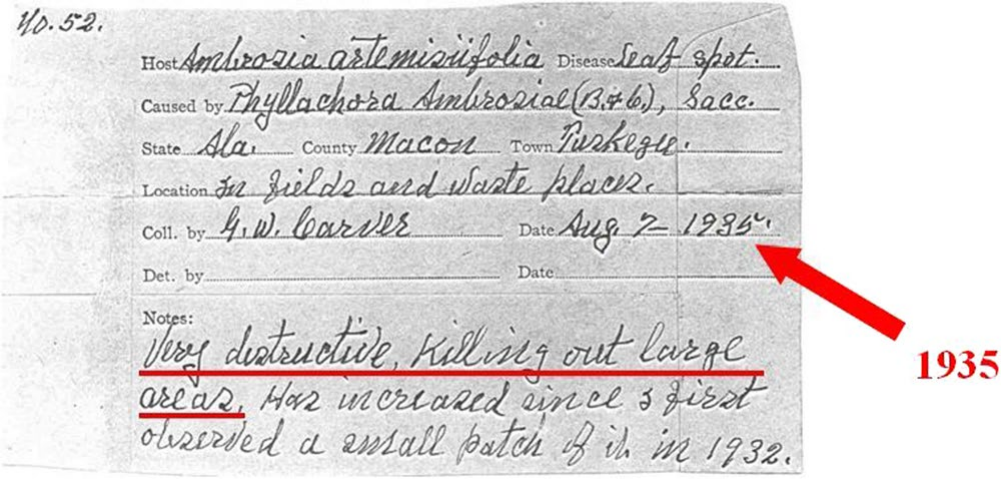
Notes attached to the *Cryptophyllachora ambrosiae* specimen BPI 636220, collected in Tuskegee, AL, USA, in 1935. According to G. W. Carver, the collector of the material, the disease was ‘Very destructive, killing out large areas. Has increased since I first observed a small patch of it in 1932’.

Perithecia and asci typical of *P. ambrosiae* were identified in two BPI specimens, BPI 636220 and BPI 636225 (Supplementary Table S3). Our attempts to amplify any nrDNA regions from perithecia identified in these two specimens were not successful, most likely because these herbarium spe cimens had been treated with insecticides at BPI, and such treatments are known to have detrimental effectson PCR amplifications^26^. The notes attached to BPI 636220 (Fig. 3) indicated that the fungus caused serious damage to *A. artemisiifolia* in Tuskegee, Alabama, USA, in 1935 and that the fungus had been found earlier at that particular site in 1932.

### Phylogenetic analyses

**Figure 4:**
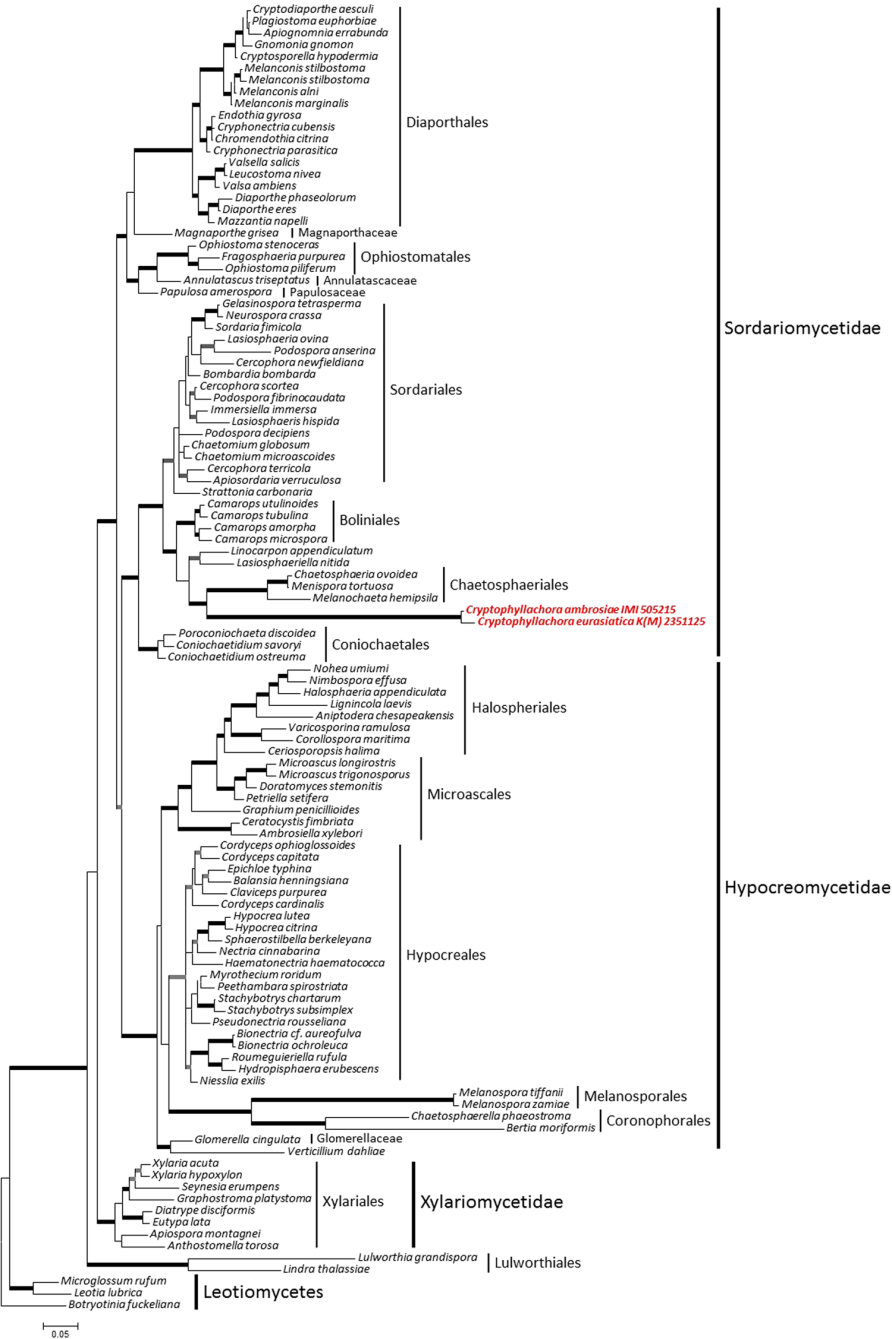
The majority consensus phylogenetic tree of Bayesian phylogenetic inference of the nrSSU and nrLSU sequences of two *Cryptophyllachora* specimens, K(M) 235112 and IMI 505215, collected in Hungary and the USA, respectively, analyzed together with a dataset of the class Sordariomycetes published by Zhang *et al*.^27^. Taxon names follow Zhang *et al*.^27^. *Botryotinia fuckeliana* was the outgroup in the analyses. Bold branches indicate strong supports: grey colour indicates Bayesian PP was not under 0.9 or Maximum Likelihood Bootstrap was not under 70% while black shows support was above those values. Bar represents 0.05 expected changes/site/branch.

Analyses of the combined nrSSU and nrLSU dataset showed that the fungi studied here belong to the subclass Sordariomycetidae in the Sordariomycetes (Fig. 4). The inclusion of the nrSSU and nrLSU sequences determined in this work did not change the general topology and relative phylogenetic positions of the clades of the Sordariomycetes shown by Zhang *et al*.^27^. The two specimens included in this analysis, K(M) 235112 and IMI 505215, originally identified as *P. ambrosiae* from Hungary and the USA, respectively, formed a long, distinct branch that was sister to the Chaetosphaeriales (Fig. 4). Nevertheless, because of the long branch lengths, specimens K(M) 235112 and IMI 505215 cannot unambiguously be considered as members of any order and are considered *incertae sedis*. The nrSSU and the nrLSU fragments sequenced in 13 specimens from Eurasia and 5 specimens from the USA (Supplementary Table S2) were identical to those of K(M) 235112 and IMI 505215, respectively, and differed from each other in a total of 26 nucleotide positions.

The second analysis based on nrLSU sequences has also shown that specimens K(M) 235112 and IMI 505215 belonged to the Sordariomycetes, but could not be assigned to any order, including the Phyllachorales. Furthermore, the two *P. ambrosiae* specimens were unambiguously separated from well-supported clades that contained morphologically similar fungi, such as *Phyllachora*, *Camarotella* and *Coccodiella* (Supplementary Fig. S3). A recently reported^28^ nrLSU sequence of *Phyllachora graminis*, the type species of the genus, was included in this analysis.

**Figure 5:**
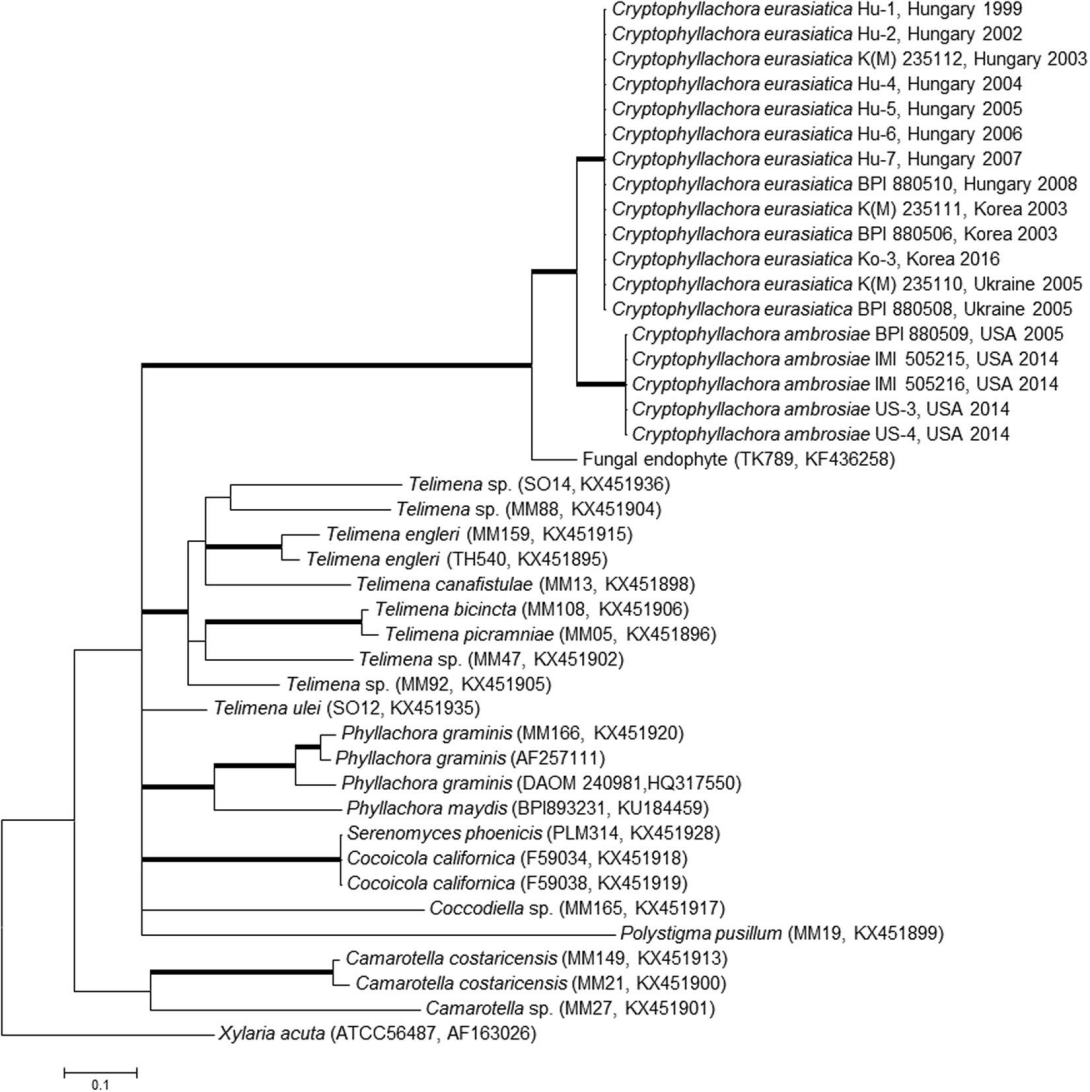
The majority consensus tree of Bayesian phylogenetic inference of the nrDNA ITS sequences of *Cryptophyllachora* specimens (Table S2) together with an ITS sequence, KF436258, from GenBank, which is currently the only sequence in GenBank with >87% similarity to *C. eurasiatica* and *C. ambrosiae* ITS sequences, and representative ITS sequences from the Phyllachorales published by Mardones *et al*.^28^. Strain or specimen designations and accession numbers are shown in parentheses for sequences obtained from GenBank. The dataset contained 42 sequences and was 664 characters long. *Xylaria acuta* strain ATCC56487 served as outgroup in the analyses. Bold branches indicates Bayesian PP support of the branch was equal or higher than 0.9. Bar represents 0.1 expected changes/site/branch.

ITS sequence analyses have also supported these results (Fig. 5). The *P. ambrosiae* specimens included in this work clustered in two well-supported clades. One contained specimens collected in Eurasia (Hungary, Korea and Ukraine), and the other had specimens from the USA, each with specimens that had identical ITS sequences (Fig. 5). The ITS sequences of the Eurasian specimens differed in 45 nucleotide positions from those determined in the specimens from the USA. Intra-sample ITS sequence polymorphism, revealed in some fungi^10,29,30^, was not detected in any specimens.

Similarity searches did not reveal any ITS sequences in public databases that were >90% similar to these specimens. The ITS sequence (KF436258) of an unidentified endophytic fungus originating from a tropical woody plant sample collected in Panama^31^ showed the highest similarity (Fig. 5). As expected from the nrSSU and nrLSU analyses (Fig. 4), *P. graminis* and other taxa of the Phyllachoraceae did not group together with *P. ambrosiae* specimens in the ITS analysis.

As phylogenetic analyses based on nrSSU, nrLSU and ITS sequences did not reveal any close relatives of specimens previously classified and identified as *P. ambrosiae*, *Cryptophyllachora* gen. nov. is introduced with two new taxa, *C. eurasiatica* sp. nov. and *C. ambrosiae* comb. nov., to accommodate the Eurasian and the North American specimens, respectively.

### Taxonomy of newly identified species

Description of *Cryptophyllachora eurasiatica* gen. et sp. nov. and *C. ambrosiae* comb. nov. (*Sordariomycetes*).

*Cryptophyllachora* L. Kiss, Kovács & R.G. Shivas, *gen*. *nov*. — Mycobank MB825649.Etymology. Refers to its morphological similarity to the genus *Phyllachora*.

Classification. *Incertae sedis*, *Sordariomycetidae*, *Sordariomycetes*.

Type species. *Cryptophyllachora eurasiatica*.

Morphologically indistinguishable from *Phyllachora* species, including the type species, *Phyllachora graminis*. Phylogenetically differentiated from other genera by unique fixed alleles in the nrSSU and nrLSU loci as shown in TreeBASE study no. 22546.

**Figure 6:**
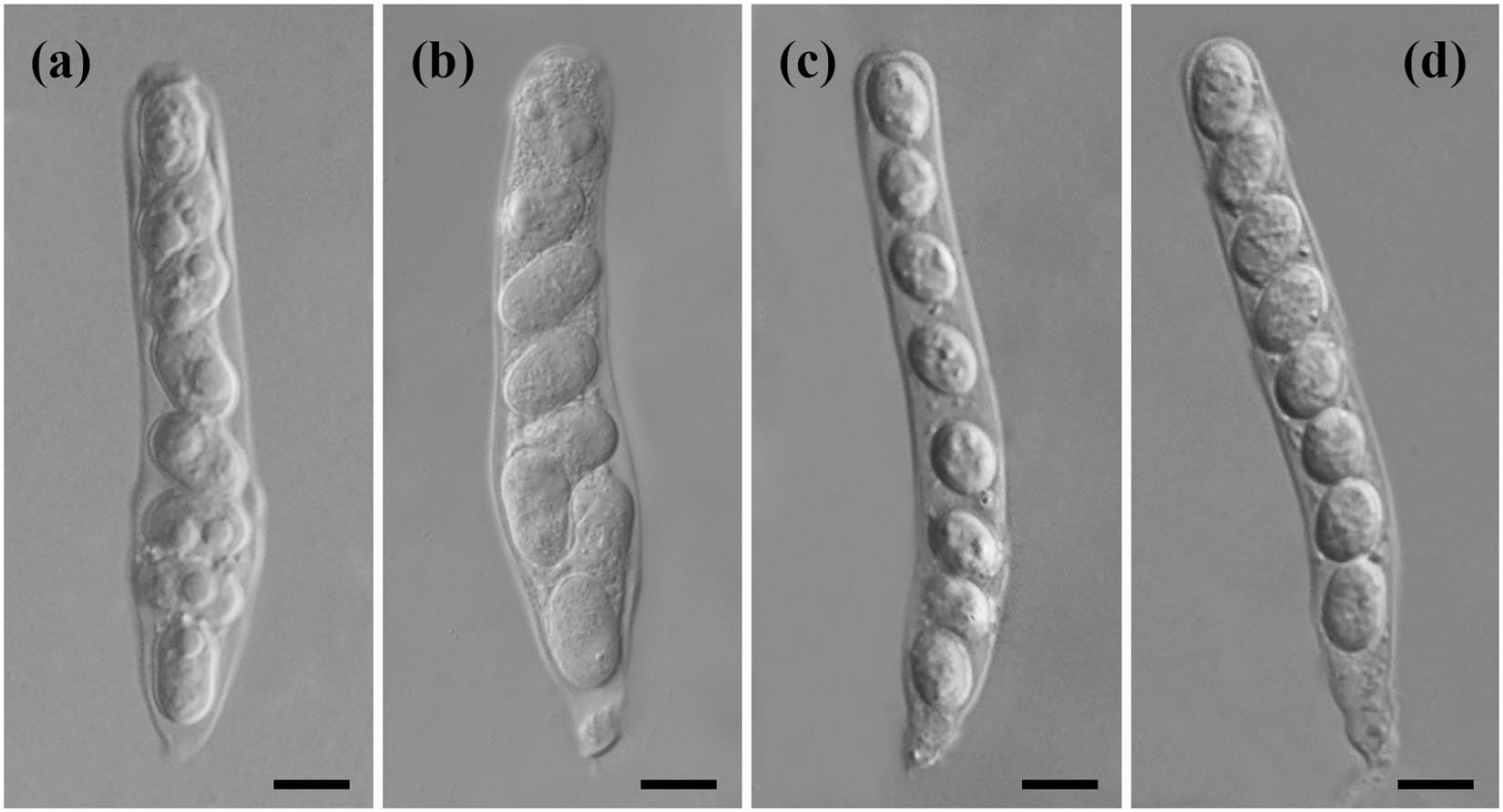
Asci of *Cryptophyllachora eurasiatica* and *C. ambrosiae*. (**a**,**b**) Asci of *C. eurasiatica* specimen K(M) 235112. (**c**,**d**) Asci of *C. ambrosiae* specimen IMI 505215. Bars = 10 µm.

*Cryptophyllachora eurasiatica* L. Kiss, Kovács & R.G. Shivas, *sp*. *nov*. — Mycobank MB 825650. Fig. 6a,b.

Etymology. Refers to its known distribution in Europe and Asia.

#### Type

Hungary, near Hatvan, 47.6694°, 19.6229°, on leaves, stems and male flowers of *Ambrosia artemisiifolia*, 9 Sept. 2003, *L. Kiss* (K(M) 235112), *ITS, SSU* and *LSU* sequences GenBank MH155435, MH155453 and MH155471, respectively.

*Spermogonia* common, pycnidial, producing filiform, hyaline, aseptate spermatia, 2–6 × 0.5–1.5 µm. *Ascomata* perithecioid, amphigenous, epiphyllous or hyphophyllous, raised, shiny, black, sparse, subglobose to globose, approx. 180–280 μm diam., immersed in the mesophyll, with a single locule, ostiole conspicuous, with periphyses in the ostiolar cavity. *Asci* broadly cylindrical in the upper two thirds, obconical in the lower third, 92–114 µm long, unitunicate, thin-walled, 8-spored, with short pedicels, formed on the basal and lateral walls of the ascoma, intermixed with hyaline, thin-walled paraphyses. *Ascospores* hyaline, unicellular, cylindrical with rounded apices, 13–22 × 8–10 µm, usually crowded towards the base of the ascus, with or without a gelatinous sheath.

#### Additional specimens examined

Hungary, Budaörs, 47.4604°, 18.8947°, on leaves, stems and male flowers of *A. artemisiifolia*, 2 Oct. 2008, *L. Kiss* (BPI 880510), *ITS, SSU* and *LSU* sequences GenBank MH155440, MH155458 and MH155476, resp.; Korea, Pocheon, 37.4517°, 127.1005°, on leaves and stems of *A. artemisiifolia*, 2 Sept. 2003, *H.D. Shin* (BPI 880505, K(M) 235111), *ITS, SSU* and *LSU* sequences GenBank MH155441, MH155459 and MH155477, resp.; Korea, Seoul, 37.3503°, 127.0126°, on leaves and stems of *A. artemisiifolia*, 6 Sept. 2003, *H.D. Shin* (BPI 880506), *ITS, SSU* and *LSU* sequences GenBank MH155442, MH155460 and MH155478, resp.; Ukraine, Dudarkiv, Boryspil district, 50.4507°, 30.9642°, on leaves and stems of *A. artemisiifolia*, 15 Sept. 2005, *V. Hayova* (BPI 880507, K(M) 235110), *ITS, SSU* and *LSU* sequences GenBank MH155444, MH155462 and MH155480, resp.; Ukraine, Kiev, Novobilychi, 50.4747°, 30.3384°, on leaves and stems of *A. artemisiifolia*, 25 Sept. 2005, *V. Hayova* (BPI 880508), *ITS, SSU* and *LSU* sequences GenBank MH155445, MH155463 and MH155481, resp.

Notes — *Cryptophyllachora eurasiatica* has localised hyphae that are both intra- and intercellular in above-ground organs of *Ambrosia artemisiifolia*. *Cryptophyllachora eurasiatica* has cylindrical ascospores with rounded ends, and usually crowded towards the base of the ascus, that differentiate it from a phylogenetically sister species, described as *Dothidea ambrosiae* Berkeley & Curtis, collected in Alabama, USA in 1876 from *A. artemisiifolia*, and re-classified below as *C. ambrosiae.* The description of *D. ambrosiae* Berkeley & Curtis mentioned that ascospores are uniseriate, which is not the case in *C. eurasiatica*. This fungus is only known to occur as a biotrophic pathogen of *A. artemisiifolia* in Eurasia (Hungary, Korea and Ukraine).

*Cryptophyllachora ambrosiae* (Sacc.) L. Kiss, Kovács, P.F. Cannon & R.G. Shivas, *comb*. *nov*. — Mycobank MB 825651. Fig. 6c,d.

*Basionym.Phyllachora ambrosiae* Sacc., *Sylloge Fungorum* **2**: 601 (1883). Synonym. *Dothidea ambrosiae* Berk. & Curtis, *Grevillea* **4**: 105 (1876), *non D. ambrosiae* Schw. (1832), *nom. illeg*.

#### Specimens examined

USA, South Carolina, unknown locality, on leaves of *Ambrosia elatior*, not dated, collector unknown (Berkeley & Curtis, North American Fungi no. 1387 (K(M) 249570, syntype of *Dothidea ambrosiae* Berk. & Curtis); USA, Alabama, unknown locality, on leaves of *Ambrosia artemisiifolia*, not dated, *Beaumont* (Berkeley & Curtis, North American Fungi no. 4668 (K(M) 249569, syntype of *Dothidea ambrosiae* Berk. & Curtis); USA, Florida, Clermont, 28.629250°, −81.695533°, on leaves and stems of *A. artemisiifolia*, 3 June 2014, *H. Müller-Schärer* (IMI 505215), *ITS, SSU* and *LSU* sequences GenBank MH155447, MH155465 and MH155483, resp.; USA, Florida, near Paradise Heights, 28.604817°, −81.547417°, on leaves and stems of *A. artemisiifolia*, 3 June 2014, *H. Müller-Schärer* (IMI 505216), *ITS, SSU* and *LSU* sequences GenBank MH155450, MH155468 and MH155486, resp.; USA, Georgia, Trifton, 31.477953°, −83.440278°, on leaves and stems of *A. artemisiifolia*, 19 Aug. 2005, *C. Nischwitz* (BPI 880509), *ITS, SSU* and *LSU* sequences GenBank MH155446, MH155464 and MH155482, resp.

Notes —*Cryptophyllachora ambrosiae* has narrowly cylindrical asci and smaller (12–17 × 7–11 µm) uniseriate, subglobose to broadly ellipsoidal ascospores that differentiates it from *C. eurasiatica*. These two species also differ by unique fixed alleles in the nrDNA ITS, SSU and LSU loci as shown in TreeBASE study no. 22546. *Cryptophyllachora ambrosiae* has no other known host plant species and has only been found in the USA (Georgia and Florida).

### Pathogenesis of *C. eurasiatica* viewed under the TEM

**Figure 7:**
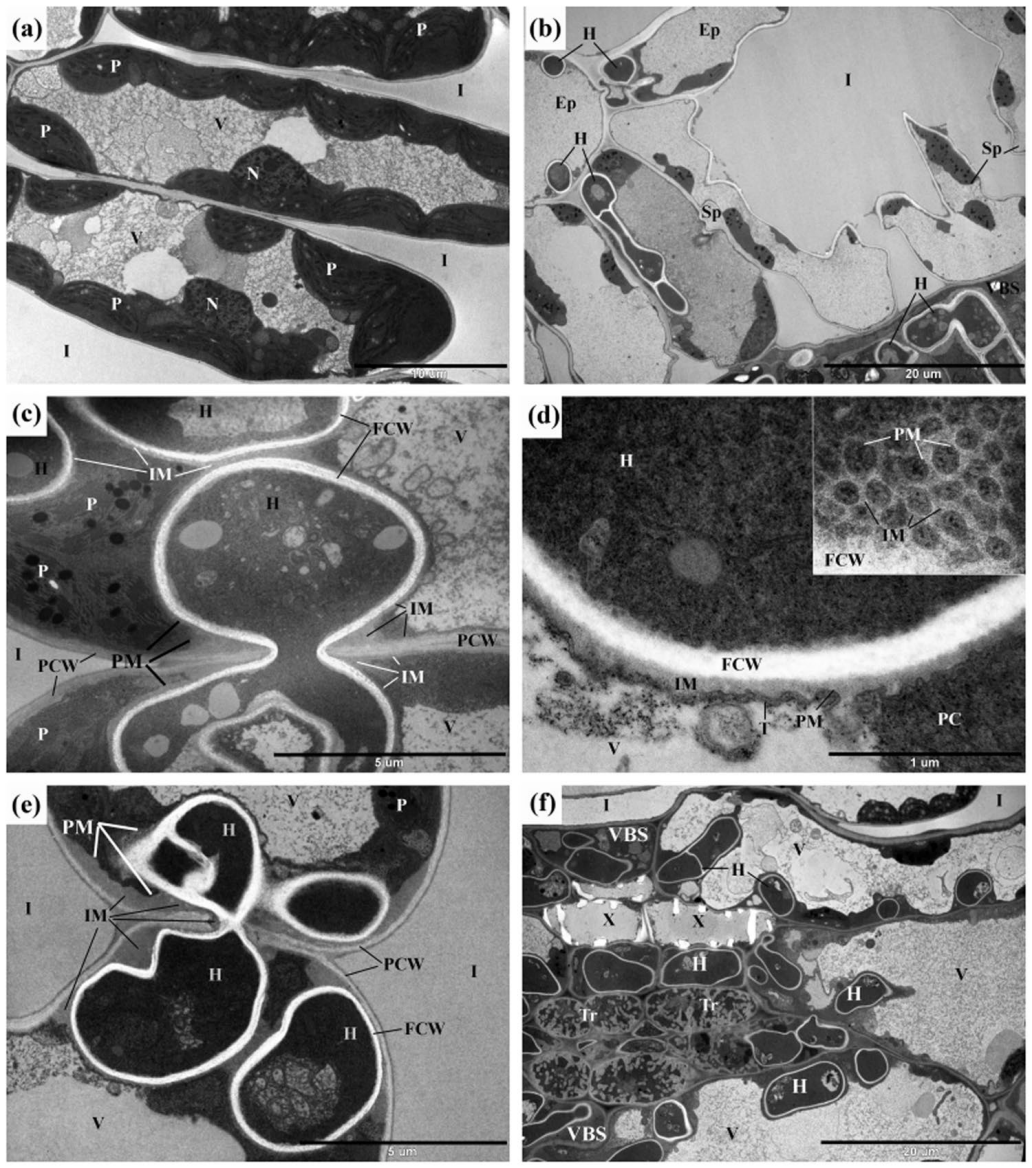
Transmission electron micrographs of hyphae of *Cryptophyllachora eurasiatica* in *Ambrosia artemisiifolia*. (**a**) Mesophyll cells of the palisade parenchyma in a symptomless leaf area 3 cm apart from leaf tissues infected with young hyphae of *C. eurasiatica*. Note in some chloroplasts (P) the enlarged and greyish plastoglobuli, the mostly curved internal membrane system, and the stroma accumulated mostly near the cell walls. (**b**) Hyphae (H) of *C. eurasiatica* in cells of the epidermis (Ep) and spongy parenchyma (Sp) as well as in the vascular bundle sheat cells (VBS). (**c**) Spread of an intracellular hypha (H) of *C. eurasiatica* from one mesophyll cell to another. Note a special interface matrix (IM) between the fungal cell wall (FCW) and the plant plasma membrane (PM). (**d**) A cross-section through the interface between an intracellular hypha (H) of *C. eurasiatica* and the host plant cell cytoplasm (PC). The surface of the interface matrix (IM) is enlarged by protuberances of interface matrix covered by the plant plasma membrane (PM). Inset: the almost longitudinal section of this part of the interface. (**e**) Large amounts of the interface matrix (IM) between the intracellular fungal cell wall (PCW) and the host plant plasma membrane (PM) and plant cell wall (PCW). (**f**) Hyphae (H) of *C. eurasiatica* in the vascular bundle sheat (VBS) cells, next to xylem (X) cells, and also in transfer (Tr) cells. Ep = epidermis; FCW = fungal cell wall; H = hyphae of *C. eurasiatica*; I = intercellular space in the mesophyll; IM = interface matrix; N = nucleus; P = plastid; PC = plant cell cytoplasm; PCW = plant cell wall; PM = plant cell plasma membrane; Sp = spongy parenchyma; T = tonoplast; Tr = transfer cell; V = vacuole; VBS = vascular bundle sheat; X = xylem.

In symptomless leaf tissues situated adjacent to young hyphae of *C. eurasiatica*, the chloroplasts in mesophyll cells were abnormal in that the plastoglobuli were enlarged and greyish in appearance. The internal membrane system was curved and stroma was accumulated in the chloroplasts mostly near the cell walls (Fig. 7a). Such changes in the chloroplast ultrastructure were interpreted as indications of biotic stress^32^.

In the yellow leaf haloes around the perithecia of *C. eurasiatica*, TEM studies revealed hyphae in the epidermal and mesophyll cells (Fig. 7b). Intracellular hyphae spread from cell to cell by penetrating the plant cell walls and entering the adjacent host cells without destroying the plant cell plasma membranes (Fig. 7c). A special matrix layer surrounding the hyphal cell walls was always present between the fungal cell wall and the intact plant cell plasma membrane (Fig. 7c–e) indicating that it is an essential component of an interface between the hyphae of *C. eurasiatica* and the colonized plant cells. This material was sometimes deposited in large quantities, especially at hyphae attached directly to the plant cell wall or penetrating the host cell wall (Fig. 7e). The surface of this interface matrix forms protruded, reticular pattern (Fig. 7d) which may enhance the short-distance transport area through this enlarged area. The presence of the interface matrix was usually not restricted to the point of penetration; sometimes it was also present along the plant cell wall and the plant cell plasma membrane to some distance from this place (Fig. 7e). The presence of such material was never observed between the wall and plasma membrane of cells of symptomless leaf tissues of *A. artemisiifolia*.

Sometimes perithecia of *C. eurasiatica* were abundant along the veins of the infected leaves (Fig. 2a) with hyphae near, and inside, vascular bundles (Supplementary Fig. S1). Ultrastructural observations also showed the presence of hyphae of *C. eurasiatica* in vascular bundle sheath cells, xylem parenchyma cells and in transfer cells (Fig. 7f).

**Figure 8:**
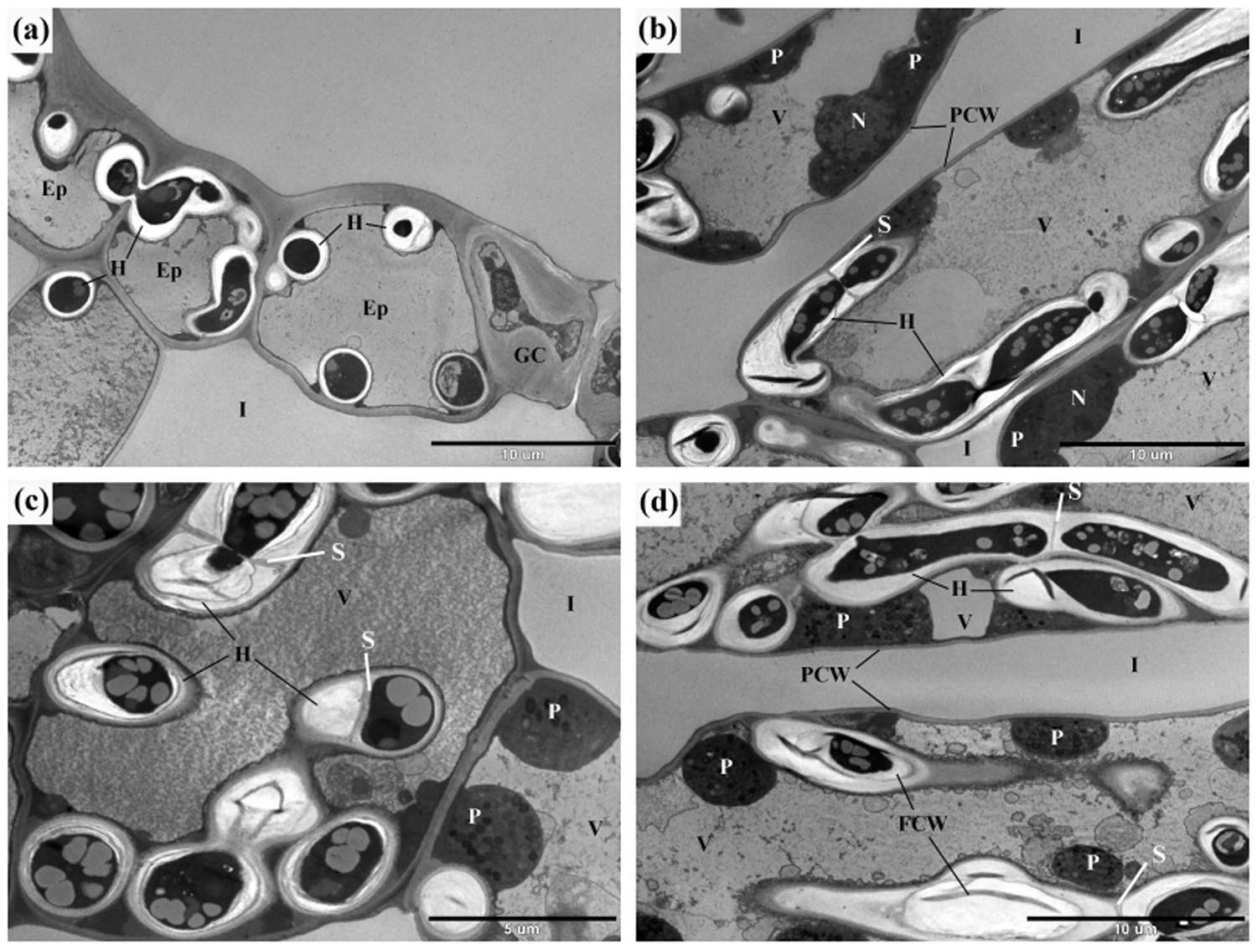
Late stages of the infection of common ragweed leaf tissues with *Cryptophyllachora eurasiatica*: sections of chlorotic, yellow leaf segments surrounding perithecia of the pathogen. (**a**) Thick-walled hyphae (H) of *C. eurasiatica* in cells of the epidermis (Ep) and mesophyll parenchyma. (**b**) Thick-walled hyphae (H) of *C. eurasiatica* in cells of the palisade parenchyma. Note the accumulation of greyish storage materials, lipid droplets in the hyphae. (**c**) Thick-walled hyphae (H) of *C. eurasiatica* in mesophyll cells. Note septa (S) in the hyphae, the accumulation of greyish storage materials in the fungal cells, and the layered structure of the thickened fungal cell walls (FCW). (**d**) At a later stage, the host plant cells’ cytoplasm is partly degraded and the ultrastructure of the plastids (P) is altered. Ep = epidermis; FCW = fungal cell wall; GC = guard cell; H = hyphae of *C. eurasiatica*; I = intercellular space; N: nucleus; P = plastid; PCW = plant cell wall; S = septum; V = vacuole.

In the crispy, senescent leaf tissues colonised by perithecia of *C. eurasiatica*, TEM revealed that the cell walls of the hyphae of *C. eurasiatica* were much thicker (Fig. 8a–d) than in the yellow leaf haloes around these areas that did not have perithecia (Fig. 7). Sometimes it was evident that the wall of the same hypha of *C. eurasiatica* thickened as the hyphal cells aged (Fig. 8a) and the fungal wall materials were deposited in layers during this thickening process (Fig. 8b–d). In conjunction, septa were blocked (Fig. 8b,c) and storage materials accumulated in the older hyphal cells of *C. eurasiatica* characterized by thicker walls (Fig. 8a–d). Although the cytoplasm of plant cells colonised by older hyphae of *C. eurasiatica* appeared degraded, some of the chloroplasts were still present there (Fig. 8b–d). The final stage of pathogenesis was characterised by plant cells containing hyphal cells that were thick-walled and full of storage materials (Fig. 8b–d).

### Study of seed transmission of *C. eurasiatica*

Hyphae of *C. eurasiatica* were not detected with light microscopy in any achenes or seed samples collected from plants infected with *C. eurasiatica*, although much of this material came from plants that were heavily infected around female inflorescences (Fig. 1f). Similarly, diagnostic PCR tests of *C. eurasiatica* did not amplify any products from these samples. Light microscopy of decolorized seedlings produced from another set of achenes did not reveal hyphae in *A. artemisiifolia* seedling tissues. Two to three month old plants grown in pots from these achenes did not show any signs of *C. eurasiatica* infection. These results indicate that seed or achene-borne transmission of *C. eurasiatica* in *A. artemisiifolia* is unlikely.

### Artificial inoculations of potted plants with ascospores of *C. eurasiatica*

In 1999 and 2008, ascospores were freshly collected from plants infected with *C. eurasiatica* in the field (Supplementary Fig. S4). In greenhouse experiments, all potted plants inoculated with ascospore suspensions of *C. eurasiatica* developed young perithecia, close to the points of inoculation 19–22 days after inoculations. Chlorotic spots were not observed on the inoculated leaves prior to the appearance of perithecia. The first symptom was always the formation of young, brownish spherical bodies on leaves that released spermatia when lightly pressed on a microscope slide. Two to four days later, these fruiting bodies released masses of ascospores in a brown mucilage (Supplementary Fig. S4). Some of the ascospores germinated in 24 h *in vitro* (Supplementary Fig. S5). In 1999, ascospores were collected from the newly formed perithecia, suspended in water, and used to infect another round of healthy, 2-month old potted *A. artemisiifolia* plants in the greenhouse. That inoculation experiment was carried out successfully three times, using ascospores collected from the potted *A. artemisiifolia* plants infected in the second experiment. In 2008, the inoculation experiment was done only once. ITS2 sequences determined in the inoculated leaf pieces with perithecia confirmed the identity of the fungus. In all cases, *C. eurasiatica* remained localized around the points of inoculations (Supplementary Fig. S2). Light microscopic observations of decolorized plant tissues were done for up to 8 weeks following inoculations, but *C. eurasiatica* hyphae were never detected in non-inoculated leaves or in the stems of the potted plants. Thus, these experiments did not indicate the development of systemic infections in the inoculated plants.

## Discussion

Although it has been highlighted many times^33–35^, it is still surprising how little is known about the multitude of unculturable fungi and other microorganisms that are associated with plants. Many fungi detected in plants as hyphae or DNA sequences, are often considered superficially as endophytes^36,37^, although their life cycle, host range and interactions with the host plants are not known. The genus *Phyllachora* sensu lato, with well over 100 described species, is a good example for such fungi. This is the first detailed analysis of the pathogenesis, disease cycle and molecular phylogeny of two fungi that were originally thought to be species of *Phyllachora*. Our results showed that a fungal species previously classified as *P. ambrosiae*, actually represented two closely related species in a distinct lineage within the Sordariomycetidae. These two fungi are described here in a new genus, *Cryptophyllachora*, as *C. eurasiatica* and *C. ambrosiae*. These two species are not closely related to *Phyllachora*, nor to the Phyllachorales, as far as we can determine by phylogenetic analyses of publicly available nrDNA sequences.

Molecular data obtained in this study were decisive in understanding the identity of these pathogens. However, the relatively low number of sample sites means that the presence of *C. eurasiatica* in North America, and *C. ambrosiae* in Eurasia, cannot be excluded. On the other hand, sample sites in the USA were over 700 km apart from each other, and those in Eurasia up to 8,000 km apart. Further, samples with identical ITS sequences were collected in different years, which strengthens the possibility that *C. eurasiatica* is restricted to Eurasia and *C. ambrosiae* to North America. *Ambrosia artemisiifolia* is native to North America and was first recorded in Europe in the late 18^th^ century^2^, and in Asia less than 100 years ago^38^. Thus, it cannot be assumed that the *C. eurasiatica* lineage repeatedly found in three distant places in Eurasia quickly evolved in the introduced *A. artemisiifolia* populations over the last 100–200 years.

This long-term study, started in 1999, has also revealed how difficult is to work with those little known, unculturable fungi which sometimes ‘disappear’ from the field, i.e. become undetectable for one or more seasons even in well explored sample sites, while causing serious epidemics in other seasons. We developed two mutually exclusive working hypotheses to explain the ‘unusual’ temporal dynamics of *Cryptophyllachora* epidemics observed in both Eurasia and the USA. The first hypothesis is that (i) *C. eurasiatica* has also evolved in North America, similar to *C. ambrosiae*, although it was not detected there in this study; (ii) it is seed-borne in native North American *A. artemisiifolia* populations, and was introduced to Eurasia as such, in the 19^th^ or 20^th^ century; and (iii) this pathogen causes systemic, mostly latent, and sometimes devastating infections in both native North American and introduced Eurasian *A. artemisiifolia* populations.

The second hypothesis is that (i) *C. eurasiatica* is an opportunistic pathogen of *A. artemisiifolia* and has alternative host(s) in Europe and/or Asia, although these host-pathogen relationships have not been identified yet; and (ii) this pathogen was not introduced to Eurasia together with *A. artemisiifolia*; instead, mainly occurs in currently unidentified host plant species which serve as sources for the rarely detected *C. eurasiatica* infections of *A. artemisiifolia*.

There could also be another, seemingly straight forward explanation for the temporal dynamics of *C. eurasiatica* epidemics, if we assumed that the inoculum (i.e., ascospores) may simply remain undetected for several years in plant litter, and may cause epidemics in *A. artemisiifolia* populations only in favourable conditions. This could be the case; however, this idea does not explain for example how *C. eurasiatica* became so widespread in Eurasia where its known host, *A. artemisiifolia*, has not been present for more than 100–150 years. Also, *A. ambrosiae* is mainly present in disturbed areas, such as agricultural fields, where it is unlikely that the leaf litter would persist for a long time, to serve as a source of inoculum for *Cryptophyllachora*.

Concerning the first hypothesis, if *C. eurasiatica* was introduced to Eurasia together with *A. artemisiifolia*, seed transmission would indeed be the most plausible way of introduction because this annual weed reproduces solely by seeds, and was probably never moved across continents as a whole plant. However, we found no evidence for seed-borne transmission of *C. eurasiatica* in this work. Furthermore, we could not detect signs of systemic infections of *A. artemisiifolia* with *C. eurasiatica* in either field conditions or greenhouse experiments.

Nevertheless, these results do not rule out the possibility of seed transmission and latent systemic infections in *C. eurasiatica*, because the detection of hyphae of different fungi in asymptomatic plant tissues is often very difficult by both microscopic methods and DNA tests. For example, in *Microbotryum* spp., extensively studied biotrophic basidiomycete fungi causing anther smut diseases, systemic infections have long been hypothesised to explain some parts of the life cycle in their perennial hosts^39^. These, however, could not be unambiguously proven for a long time, because DNA tests did not detect fungal structures in asymptomatic stems and the visualization of hyphae in dormant and young host plant tissues was very difficult^17^. In *Botrytis* spp., mostly known as destructive necrotrophic plant pathogens^40^, it has long been revealed that some strains are seed-borne, and cause latent, systemic infections^40–43^. However, it was not until recently that it has become clear how frequently *Botrytis* spp. can exist in asymptomatic seeds and host plant tissues, during the whole life cycle of their host plants, and that they do not need to cause disease in order to complete their life cycles^44^. Recently, the hidden existence of some truly endophytic fungi was explained by the formation of a little known, and difficult to detect, protoplast phase in host plant tissues^45^. Therefore, seed transmission and systemic infection cannot be completely excluded in little studied fungi such as *C. eurasiatica*.

On the other hand, an anther smut fungus, *M. lychnidis-dioicae*, infecting *Silene latifolia*, provides a good example that even a non-agricultural, biotrophic, and insect-vectored plant pathogen, that is neither transmitted by seeds nor persistent in the environment, and suffering from a very strong genetic bottleneck, can successfully move from one continent to another, and establish in its introduced host plant populations^46^. Thus, seed transmission may not be the only way of inter-continental movement even for highly specialized, biotrophic fungal plant pathogens with restricted host ranges.

The second hypothesis proposes that there are alternative hosts of *C. eurasiatica* in Eurasia. If this was the case, the *C. eurasiatica* lineage repeatedly found in three distant places in Eurasia would have co-evolved with its alternative host(s), long before *A. artemisiifolia* was introduced from North America. However, there are no nrDNA sequences available in databases that are similar to those of *C. eurasiatica*, in spite of comprehensive metagenomics and microbial diversity studies. Candidates for alternative hosts are most likely to be found in the Asteraceae; however, no *Phyllachora* species are known to occur on any asteraceous plant species but *A. artemisiifolia* in Europe.

Our inoculation experiments revealed that the disease cycle of *C. eurasiatica* can be completed in *A. artemisiifolia* plants by ascosporic infections, and these infections can cause repeated cycles of disease in *A. artemisiifolia* populations within a single season. Also, *C. eurasiatica* is apparently adapted to survive in the infected, and dried, *A. artemisiifolia* tissues, although the viability of the thick-walled survival structures produced in senescent plant tissues, and revealed by TEM, were not demonstrated in this work.

The initial observations of a considerable damage caused by *C. eurasiatica* in Hungary^22^ and Ukraine^23^ by destroying the male inflorescences of *A. artemisiifolia* in the field suggested that this fungus may be a cryptic friend of humans allergic to the pollen of this invasive weed. These observations have also suggested that plant diseases may sometimes have an impact on human pollen allergies, which is still a very little explored topic. The results obtained in this work may trigger further studies that could explore the potential of *Cryptophyllachora* spp. as classical or inundative BCAs of *A. artemisiifolia*.

## Materials and Methods

### Surveys and specimens

Since the mid-1990s, there have been regular surveys for fungal pathogens of *A. artemisiifolia* at sites in Hungary, Ukraine and Korea. The sizes of the surveyed *A. artemisiifolia* populations in these countries have varied with time due to changes in agricultural practices, the crops cultivated, landscape management, and plant succession in abandoned fields. Supplementary Table S1 shows the characteristics of the field survey sites in Hungary, Korea and Ukraine that were regularly monitored for the presence of *P. ambrosiae*, over a period of at least five years. In North America, three field surveys were conducted, one in the USA and Canada in 2003^47^, one in Georgia (in 2005) and the last one in Florida (in 2014). Plants infected with *P. ambrosiae* were identified in the field based on the visible presence of perithecia on leaves, stems, and male and female inflorescences (Figs 1, 2a). Some parts of the infected plants were preserved as herbarium specimens and deposited at Herbarium IMI (Royal Botanic Gardens, Kew, U.K.) and BPI (Supplementary Table S2).

In addition, 13 specimens of *P. ambrosiae* available at BPI, and collected from 1905–1962, were also examined (Supplementary Table S3). Transmission electron microscopy studies, artificial inoculations in greenhouse experiments and seed transmission studies were completed with fresh specimens of *C.* *eurasiatica* and *A. artemisiifolia* collected in Hungary.

### Light microscopy

The morphology of *C. eurasiatica* was examined by bright field and differential interference contrast (DIC) microscopic examination of fresh, infected plant tissues macerated in a droplet of water. To confirm the identity of the pathogen, nrDNA ITS2 sequences were determined in these samples (see below). Herbarium specimens were microscopically examined after being boiled in lactic acid. The spread of the fungus in plant tissues was microscopically examined in decolorized leaf segments (Supplementary Fig. S2) fixed in Carnoy’s solution (6:3:1 ethanol:chloroform:glacial acetic acid, by volume) to remove chlorophyll.

### Transmission electron microscopy (TEM)

To prepare samples for TEM, infected leaf segments approx. 2 × 2 mm, were fixed in 2% glutaraldehyde for 3 h and in 1% osmium tetroxide for 2 h (both fixatives were dissolved in 0.1 M K-Na-phosphate buffer, pH 7.2) and embedded in Durcupan resin after dehydration in an ethanol series ended with propylene oxide. Semi-thin sections (1 μm) for light microscopy were prepared with a Microm HM 360 microtome (Microm, Walldorf, Germany) and stained with toluidine blue. Ultra-thin sections (70 nm) were cut with a Reichert Ultracut E ultramicrotome (Leica Microsystems, Vienna, Austria) and mounted on uncoated Cu/Pd grids (Polysciences, Warrington, PA, USA), and stained with 2% uranyl acetate dissolved in methanol for 4 min and lead citrate for 6 min. Sections were examined with a Hitachi 7100 transmission electron microscope (Hitachi, Tokyo, Japan) at 75 kV.

### DNA extraction, amplification and sequencing

To obtain DNA of *C. eurasiatica* free from contamination by plant, microbial or other sources, the initial extractions were made from single fresh mucilaginous droplets of ascospores (Supplementary Fig. S4) released from ascomata of sample Hu-1. These droplets were collected with sterile glass needles in sterile 1.5 ml plastic tubes under a dissecting microscope. Small amounts of ascospores were examined under a light microscope with magnifications up to 1000x to ensure that they did not contain contaminant cells or cell fractions other than *C. eurasiatica* ascospores. DNA was extracted using the Chelex method and the ITS region was amplified in a semi-nested PCR, and sequenced with universal and newly designed primers as described previously^48^.

The ITS sequence determined in this way, deposited in GenBank as MH155433, was used to design the *C. eurasiatica*-specific ITS primers P-F (5′-CAGATCTCCGTGAGCCATCGAA-3′) and P-R (5′-CCTGGCGTAGTGATTATAGCTC-3′). These were used in all the subsequent PCRs done with DNA samples extracted from fresh or dried plant tissues containing perithecia. These DNA extractions were done with Qiagen DNeasy Plant Mini Kit (Qiagen GmbH, Hilden, Germany) or the CTAB method^26^ with prolonged centrifugation lengths (20 min) and overnight isopropanol precipitation.

The ITS region was amplified from these *C. eurasiatica* DNA samples in two steps. Primers ITS1F^49^ and P-R were used to amplify a DNA sequence containing ITS1, 5.8S nrDNA and a 3′ part of ITS2 of the ITS region of the fungus. Another DNA fragment, spanning the complete ITS2 sequence, was amplified with primers P-F and ITS4^50^. The PCR protocol consisted of an initial denaturation at 94 °C for 5 minutes; 35 cycles of denaturation for 30 sec at 94 °C, primer annealing for 30 sec and extension at 72 °C for 40 sec, followed by a final extension for 10 min. Annealing temperatures were 52 °C for the ITS1F/P-R and 55 °C for the P-F/ITS4 primer pairs.

A DNA fragment of the fungus that encompassed the 3′ region of 18S nrDNA (nrSSU), as well as the complete ITS1 and 5.8S sequences and a 3′ part of ITS2, was amplified with primers NS5 or alternatively NS3^50^ and P-R. Another DNA fragment spanning ITS2 and a 5′ part of 28 S nrDNA (nrLSU) was amplified with primers P-F and LR5^51^. PCRs consisted of an initial denaturation at 95 °C for 5 min, denaturation for 30 sec at 95 °C, annealing for 30 sec and 1.5 min extension at 72 °C for 35 cycles, followed by a 7 min final extension. Annealing temperatures were 55 °C for the nrSSU and 50 °C for the nrLSU fragment amplifications.

In all PCRs, DreamTaq DNA Polymerase (Thermo Fisher Scientific, Waltham, USA) was used in a final volume of 20 μl, with 500 nM primer (Sigma) concentration and 2 μl of total DNA, which was sometimes diluted 10x or 100x before use to improve amplification reliability. Each PCR was done in parallel in three tubes to eliminate sequencing errors^29,52^. PCR products were sequenced using BigDye Terminator v3.1 Cycle Sequencing Kit (Thermo Fischer Scientific) with the primers used for the respective PCRs. In addition, primers NS6, NS7 and NS8^50^, as well as LR0R, LR3 and LR3R^51^ were also used for sequencing the nrSSU and nrLSU fragments, respectively. Representative DNA sequences were deposited in GenBank (Supplementary Table S2).

### Phylogenetic analyses

Manually verified electrophoregrams were used to generate DNA sequences with the Staden package^53^. Three datasets were analysed, (i) ITS sequences together with the most similar ITS sequence (KF436258) in NCBI GenBank, found with a BLAST search, and coming from an unidentified fungal endophyte^31^, as well as representative ITS sequences from the Phyllachorales^28^; (ii) nrSSU and nrLSU sequences determined in this study, which were combined in a single dataset, and analysed together with a comprehensive dataset compiled by Zhang *et al*.^27^ for the Sordariomycetes; and (iii) a dataset of nrLSU sequences (approx. 700 bp) determined in this study, and analysed together with representative nrLSU sequences of the Phyllachorales reported earlier^27,28^.

Sequences were aligned with MAFFT v. 7^54^ by the auto option on the webserver. Bayesian (BI) analyses were performed with MrBayes 3.1.2^55,56^. The GTR + G model was applied for (i) ITS; (ii) partial nrLSU; and (iii) nrSSU and nrLSU sequences divided into two partitions. Four Markov chains were run for 10,000,000 generations, sampling every 1000 steps, and with a burn in at 4000 sampled. The convergence of the MCMC Bayesian phylogenetic inferences was checked by AWTY online^57^. Maximum likelihood (ML) phylogenetic analyses of the nrSSU and nrLSU dataset was carried out with RAxML^58^ using the raxmlGUI^59^ with GTRGAMMA. Bootstrap analysis with 1000 replicates was used to test the support of the branches of the best tree gained. Phylogenetic trees were visualized and edited in MEGA6^60^.

### Seed transmission

More than 1000 achenes were collected from *A. artemisiifolia* plants infected with *P. ambrosiae* in the field in Hungary in 1999, 2003, 2006, 2007 and 2008, in autumn, when achenes are mature. Whenever possible, these achenes were collected from plants with abundant perithecia around the former female inflorescences (Fig. 1f). Approximately half of the achenes were sectioned under a dissecting microscope up to one week after collection. Seed tissues were microscopically examined for the presence of seed-borne hyphae as described above. In addition, DNA was extracted from mixtures of 10 sectioned seeds and PCRs were run with the primer pair P-F/ITS4 as described above, to check the presence of *P. ambrosiae* in these samples. A positive DNA sample, as well as a DNA-free negative control were included in PCRs.

The remainder of the achenes were kept at 4–6 °C for 4 months, then germinated on wet filter paper discs in plates exposed to 16 h daily illumination. About half of the seedlings were fixed in either Carnoy’s solution or a 1:1 mixture of acetic acid and absolute ethanol for 2 to 5 d, then rinsed with water, and examined for the presence of seed-borne hyphae with light microscopy. The rest of the seedlings were planted in pots filled with garden soil, one seedling per pot. Pots were kept for 2–3 months in a greenhouse at a day/night temperature regime of 20–25/13–15 °C under natural light conditions. Plants produced in this way were monitored weekly for ascomata on their aerial surfaces and at the end of the experiments asymptomatic leaf and stem segments of 2–3 month old plants were removed from pots, rinsed with water, decolorized in Carnoy’s solution, and examined by light microscopy.

### Inoculation experiments

The first experiment was done in October 1999 with five 2-month old potted *A. artemisiifolia* plants, grown in an experimental greenhouse from achenes collected in Hungary in 1996. An aqueous ascospore suspension, containing 5 × 10^5^ ascospores per ml, was prepared from mucilaginous droplets released by perithecia on field-collected leaves (Supplementary Fig. S4), and collected with glass needles under a dissecting microscope. Leaves of potted plants were inoculated by pipetting 100 µl droplets of the ascospore suspension close to the main veins, one droplet per leaf, as described earlier^22^. Inoculated plants were placed in a moist chamber for 48 h, then kept in a greenhouse at 20–25 °C under natural light conditions, and observed for at least 8 weeks after inoculations. Five non-inoculated plants served as controls. Ascospore germination (Supplementary Fig. S5), as a proof of ascospore viability, was verified after 24 h incubation of 20 µl suspension used for inoculations on sterile cellophane placed on 1.5% water agar medium^61^. The experiment was repeated in November 1999, using ascospores collected from potted plants inoculated in October that year (Supplementary Fig. S4); then in December 1999, with ascospores from plants inoculated in November 1999; and then the same experiment was done in October 2008 with ascospores collected from the field that time.

## Electronic supplementary material

Supplementary Figures S1-S5 and Tables S1-S3
