# Supplementary material for: Deciphering the biology of Cryptophyllachora eurasiatica gen. et sp. nov., an often cryptic pathogen of an allergenic weed, Ambrosia artemisiifolia
Source: Sci Rep. 2018 Jul 17;8:10806. doi: 10.1038/s41598-018-29102-5 (PMC6050288; doi:10.1038/s41598-018-29102-5)
Supplement: Supplementary file 1 — Supplementary Figures S1-S5 and Tables S1-S3 [file 41598_2018_29102_MOESM1_ESM.pdf]

SUPPLEMENTARY MATERIAL

Deciphering the biology of *Cryptophyllachora eurasiatica* gen. et sp. nov., an often cryptic pathogen of an allergenic weed, *Ambrosia artemisiifolia*

Levente Kiss[1,2*], Gábor M. Kovács[2,3], Károly Bóka[3], Gyula Bohár[4], Krisztina Varga Bohárné[4], Márk Z. Németh[2], Susumu Takamatsu[5], Hyeon-Dong Shin[6], Vera Hayova[7], Claudia Nischwitz[8], Marion K. Seier[9], Harry C. Evans[9], Paul Cannon[10], Gavin James Ash[1], Roger G. Shivas[1], and Heinz Müller-Schärer[11]

[1]University of Southern Queensland, Centre for Crop Health, Toowoomba, Qld 4350, Australia
[2]Plant Protection Institute, Centre for Agricultural Research, Hungarian Academy of Sciences (MTA-ATK), Budapest, H-1525, Hungary
[3]Eötvös Loránd University, Institute of Biology, Department of Plant Anatomy, Budapest, H-1117, Hungary
[4]Biovéd 2005 Ltd., Kemenestaródfa, H-9923, Hungary
[5]Mie University, Graduate School of Bioresources, Tsu, 514-8507, Japan
[6]Korea University, Division of Environmental Science and Ecological Engineering, Seoul, 02841, Korea
[7]National Academy of Sciences of Ukraine, M.G. Kholodny Institute of Botany, Kyiv, 01004, Ukraine
[8]Utah State University, Department of Biology, Logan, UT 84322, USA
[9]CABI Europe-UK, Egham, Surrey, TW20 9TY, United Kingdom
[10]Royal Botanic Gardens, Jodrell Laboratory, Mycology Section, Kew, TW9 3AB, United Kingdom
[11]University of Fribourg, Department of Biology/Ecology & Evolution, Fribourg, CH-1700, Switzerland

*Corresponding author (Email: Levente.Kiss@usq.edu.au)

This file contains all the supplementary figures (Figs. S1-S5) and supplementary tables (Tables S1-S3) of this work.

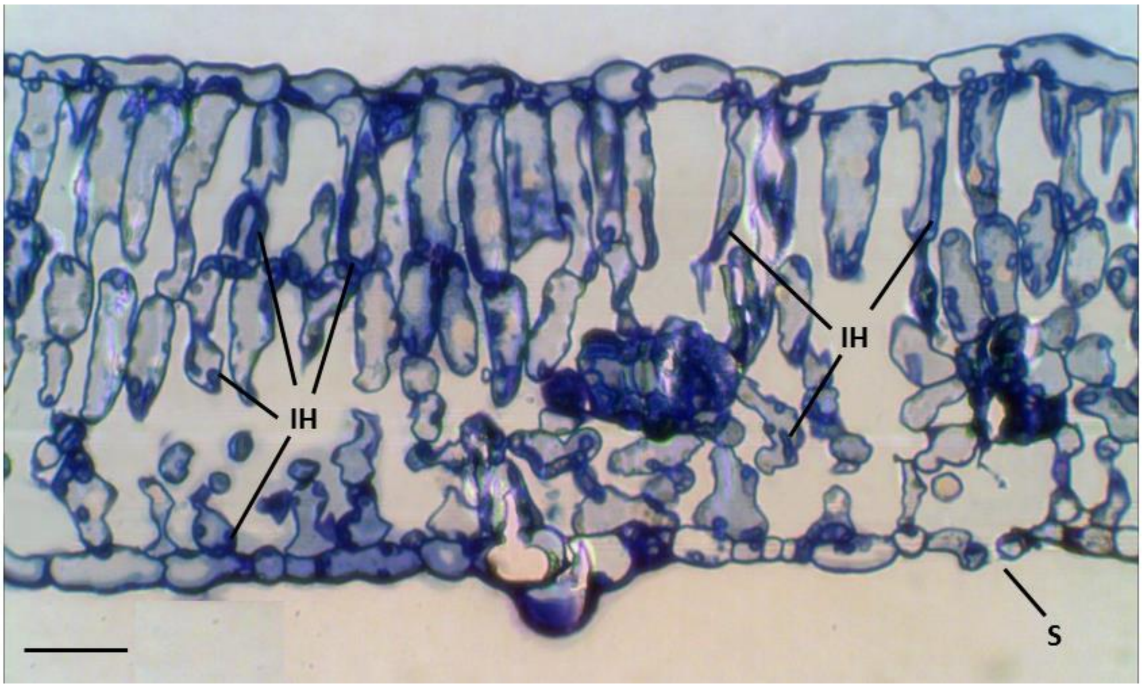

Supplementary Figure S1. A semi-thin section of a common ragweed leaf infected with *Cryptophyllachora eurasiatica*, stained with toluidine blue. Intracellular hyphae (IH) are visible in the epidermal cells, as well as in the mesophyll. S = stoma. Bar = 50 µm.

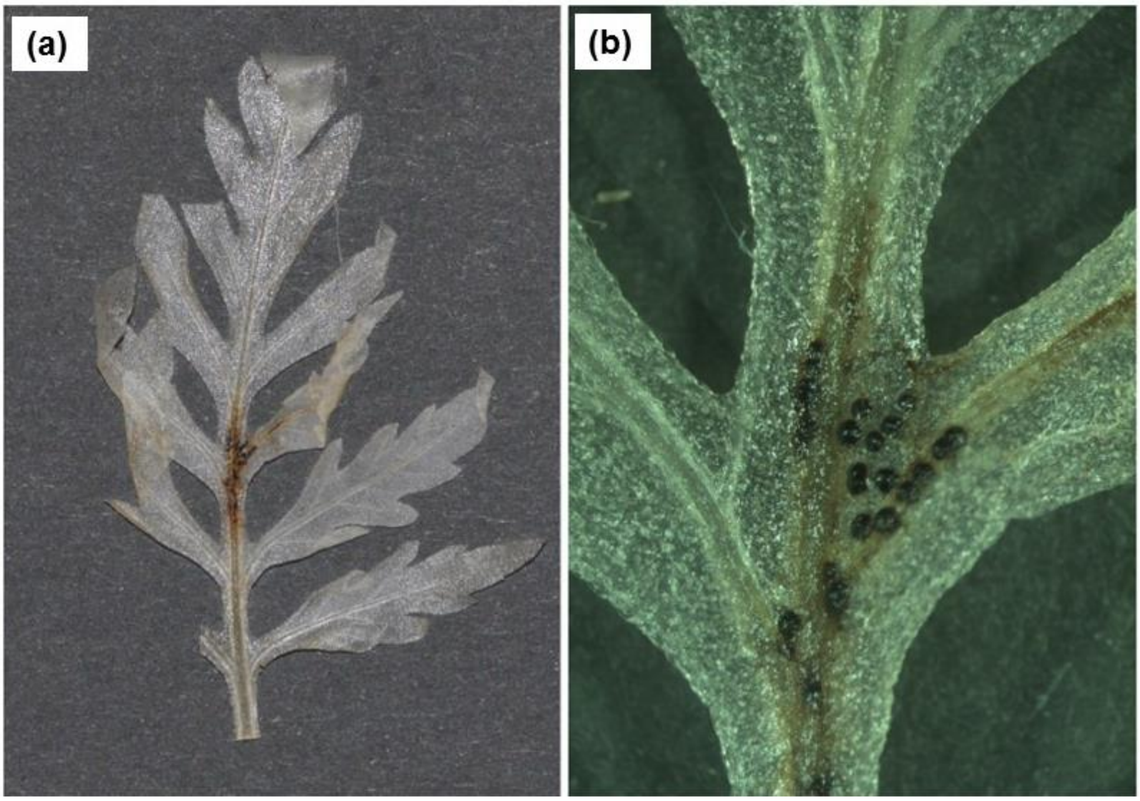

Supplementary Figure S2. A leaf of a potted common ragweed plant decolorized in Carnoy's solution one month following artificial inoculation with a *Cryptophyllachora eurasiatica* ascosporic suspension. Mature perithecia are visible around the point of inoculation, but not elsewhere. **(a)** The whole leaf. **(b)** A close-up of the leaf surface with perithecia.

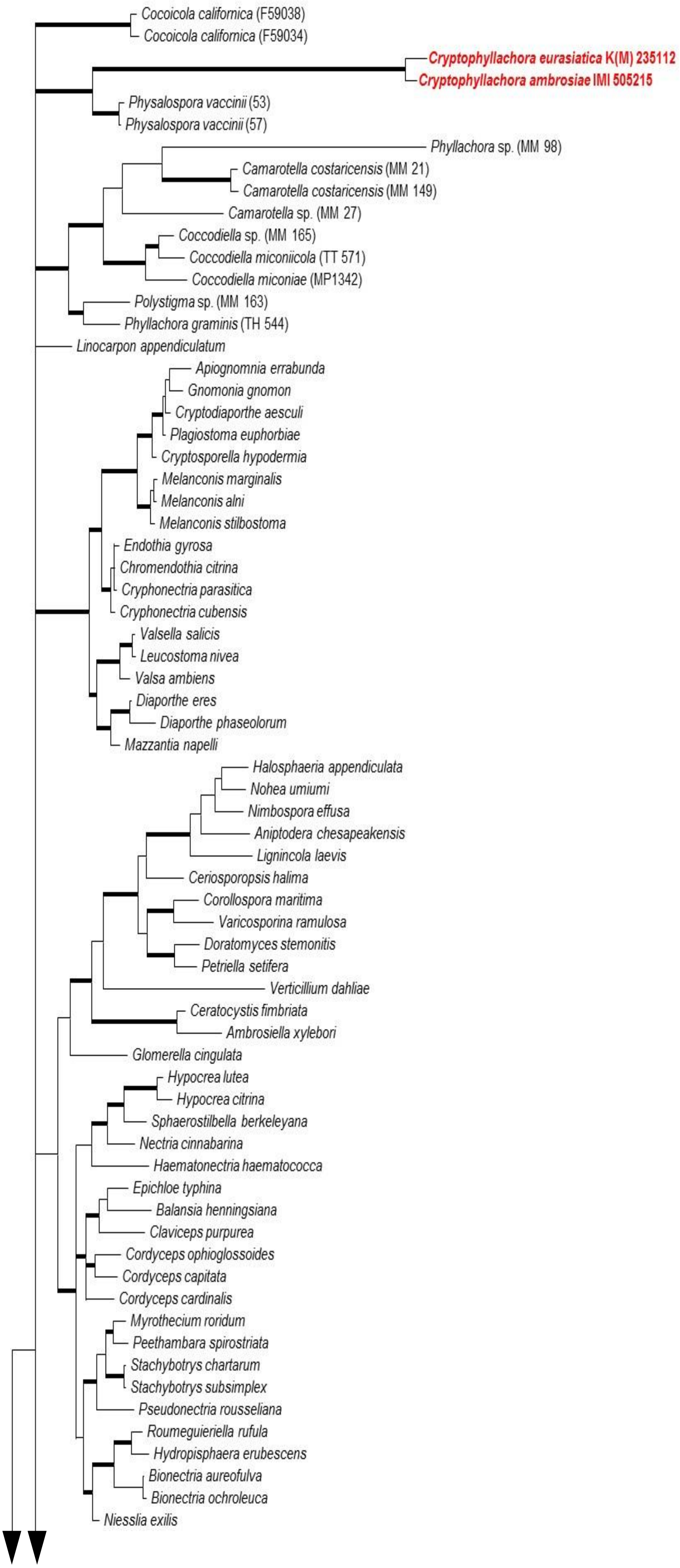
Cocoicola californica (F59038)
Cocoicola californica (F59034)
Cryptophyllachora eurasiatica K(M) 235112
Cryptophyllachora ambrosiae IMI 505215
Physalospora vaccinii (53)
Physalospora vaccinii (57)
Phyllachora sp. (MM 98)
Camarotella costaricensis (MM 21)
Camarotella costaricensis (MM 149)
Camarotella sp. (MM 27)
Coccodiella sp. (MM 165)
Coccodiella miconiicola (TT 571)
Coccodiella miconiae (MP1342)
Polystigma sp. (MM 163)
Phyllachora graminis (TH 544)
Linocarpon appendiculatum
Apiognomnia errabunda
Gnomonia gnomon
Cryptodiaporthe aesculi
Plagiostoma euphorbiae
Cryptosporella hypodermia
Melanconis marginalis
Melanconis alni
Melanconis stilbostoma
Endothia gyrosa
Chromendothia citrina
Cryphonectria parasitica
Cryphonectria cubensis
Valsella salicis
Leucostoma nivea
Valsa ambiens
Diaporthe eres
Diaporthe phaseolorum
Mazzantia napelli
Halosphaeria appendiculata
Nohea umiumi
Nimbospora effusa
Aniptodera chesapeakensis
Lignincola laevis
Ceriosporopsis halima
Corollospora maritima
Varicosporina ramulosa
Doratomyces stemonitis
Petriella setifera
Verticillium dahliae
Ceratocystis fimbriata
Ambrosiella xylebori
Glomerella cingulata
Hypocrea lutea
Hypocrea citrina
Sphaerostilbella berkeleyana
Nectria cinnabarina
Haematonectria haematococca
Epichloe typhina
Balansia henningsiana
Claviceps purpurea
Cordyceps ophioglossoides
Cordyceps capitata
Cordyceps cardinalis
Myrothecium roridum
Peethambara spirostriata
Stachybotrys chartarum
Stachybotrys subsimplex
Pseudonectria rousseliana
Roumeguieriella rufula
Hydropisphaera erubescens
Bionectria aureofulva
Bionectria ochroleuca
Niesslia exilis

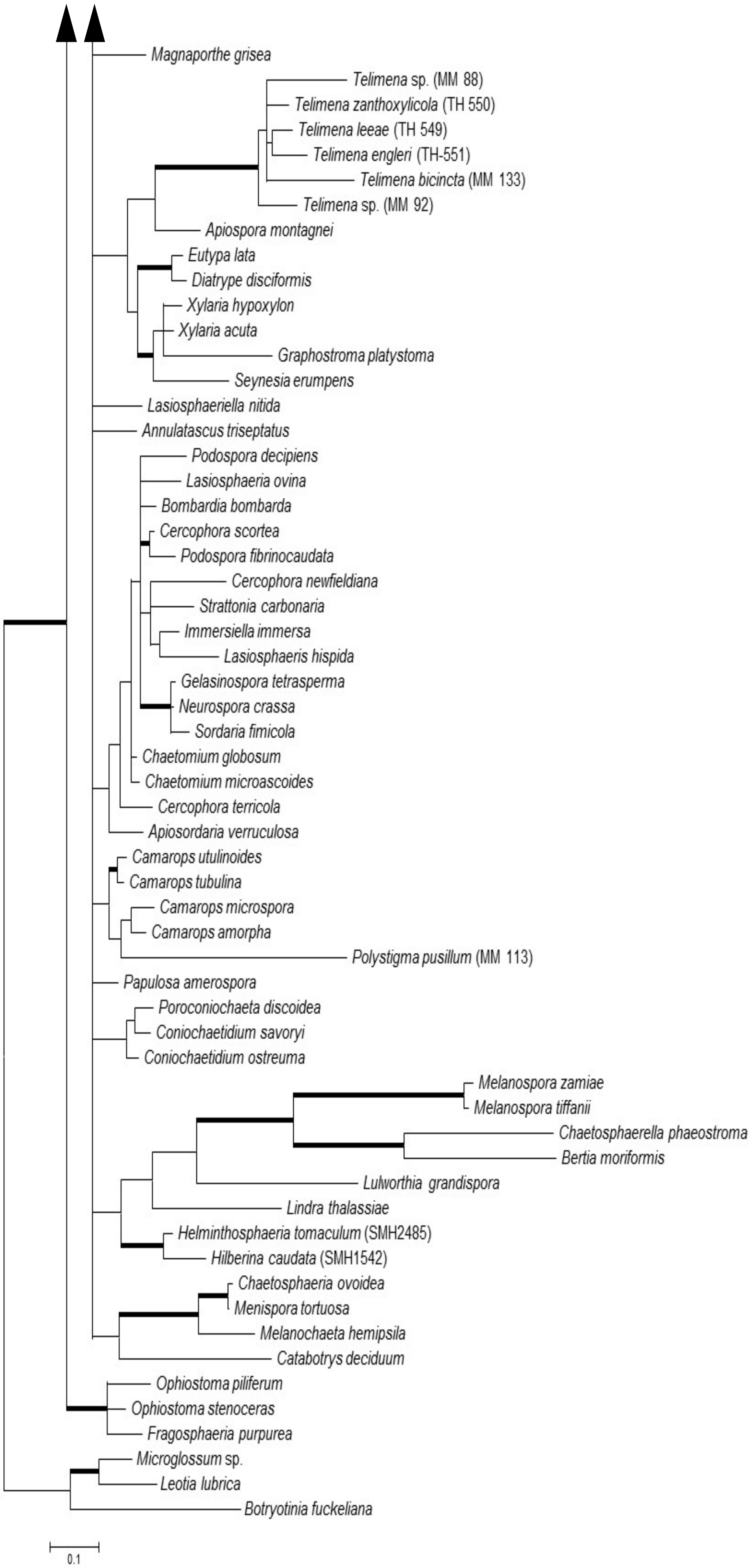
Magnaporthe grisea
Telimena sp. (MM 88)
Telimena zanthoxylicola (TH 550)
Telimena leeae (TH 549)
Telimena engleri (TH-551)
Telimena bicincta (MM 133)
Telimena sp. (MM 92)
Apiospora montagnei
Eutypa lata
Diatrype disciformis
Xylaria hypoxylon
Xylaria acuta
Graphostroma platystoma
Seynesia erumpens
Lasiosphaeriella nitida
Annulatascus triseptatus
Podospora decipiens
Lasiosphaeria ovina
Bombardia bombarda
Cercophora scortea
Podospora fibrinocaudata
Cercophora newfieldiana
Strattonia carbonaria
Immersiella immersa
Lasiosphaeris hispida
Gelasinospora tetrasperma
Neurospora crassa
Sordaria fimicola
Chaetomium globosum
Chaetomium microascoides
Cercophora terricola
Apiosordaria verruculosa
Camarops utulinoides
Camarops tubulina
Camarops microspora
Camarops amorpha
Polystigma pusillum (MM 113)
Papulosa amerospora
Poroconiochaeta discoidea
Coniochaetidium savoryi
Coniochaetidium ostreuma
Melanospora zamiae
Melanospora tiffanii
Chaetosphaerella phaeostroma
Bertia moriformis
Lulworthia grandispora
Lindra thalassiae
Helminthosphaeria tomaculum (SMH2485)
Hilberina caudata (SMH1542)
Chaetosphaeria ovoidea
Menispora tortuosa
Melanochaeta hemipsila
Catabotrys deciduum
Ophiostoma piliferum
Ophiostoma stenoceras
Fragosphaeria purpurea
Microglossum sp.
Leotia lubrica
Botryotinia fuckeliana
0.1

Supplementary Figure S3. The majority consensus tree of Bayesian phylogenetic inference of the nrLSU sequences of two *Cryptophyllachora* specimens, K(M) 235112 and IMI 505215, collected in Hungary and the USA, respectively, analyzed together with most of the sequences used by Zhang *et al.* (2006), and included in our analysis of the combined nrLSU and nrSSU dataset (see Fig. 5), and, in addition, with representative nrLSU sequences of the Phyllachorales published recently by Mardones *et al.* (2017). Taxon names follow the original publications. The dataset contained 128 sequences and was 691 characters long. *Botryotinia fuckeliana* served as outgroup in the analyses. Bold branches indicate that Bayesian PP support was equal or higher than 0.9. Bar represents 0.1 expected changes/site/branch.

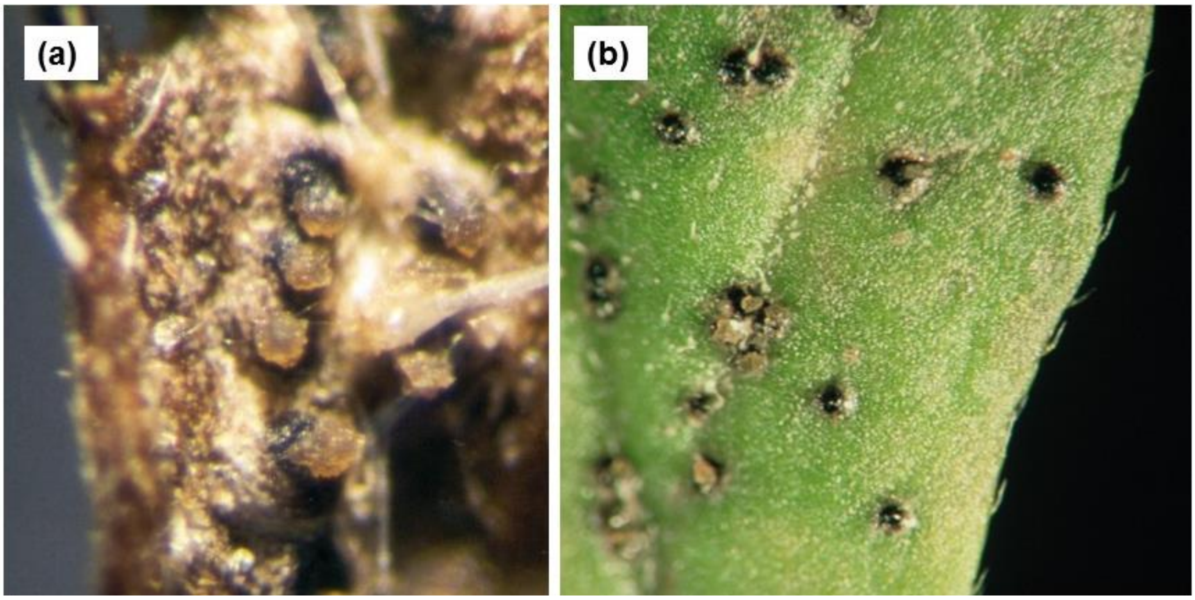

Supplementary Figure S4. Ascospore release from *Cryptophyllachora eurasiatica* perithecia. **(a)** Release of ascospores in a brownish mucilaginous material from perithecia found on an already dry, crispy part of a common ragweed plant collected in the field. **(b)** Ascospore release from perithecia produced in a potted plant one month following artificial inoculation with a *C. eurasiatica* ascosporic suspension.

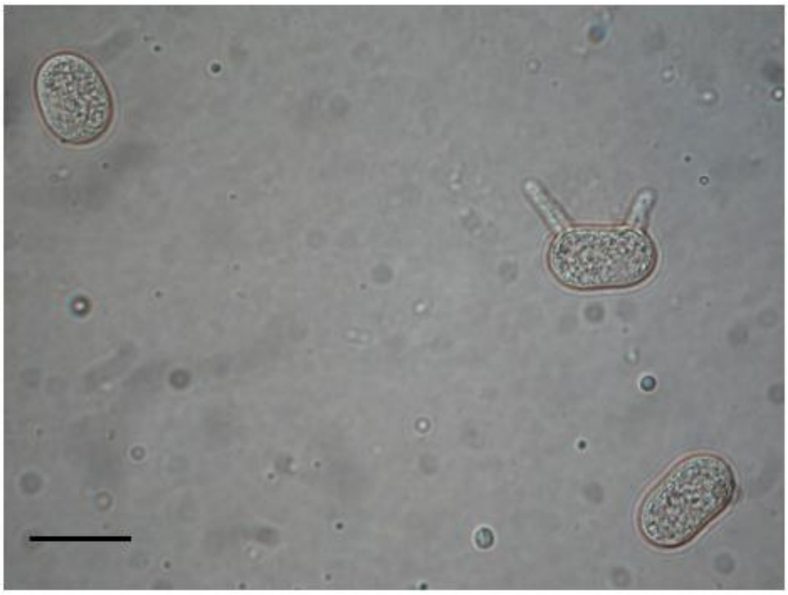

Supplementary Figure S5. A germinating, and two non-germinating ascospores of *Cryptophyllachora eurasiatica* following 24 hour incubation of an aqueous ascospore suspension on cellophane placed on water agar. Bar = 15 µm.

Supplementary Table S1. Characteristics of the Eurasian field survey sites where *Ambrosia artemisiifolia* populations were monitored for at least five consecutive years for symptoms of fungal diseases.

Locality	Habitat description	Coordinates	Duration of field surveys	Number of years when *C. eurasiatica* was detected / Duration of the survey
Hungary				
Kál	Roadside	47.7200, 20.2825	2004-2008	2/5
Biatorbágy	new residential area construction	47.4789, 18.8153	2005-2015	3/11
Etyek	roadside	47.4508, 18.7481	1999-2004	1/6
Hatvan	roadside	47.6694, 19.6229	2003-2007	2/5
Esztergom	roadside	47.7324, 18.7385	2002-2006	1/5
Kemenestaródfa	agricultural field	46.9959, 16.5252	2008-2013	2/6
Budaörs	agricultural field	47.4604, 18.8947	2008-2014	2/7
Korea				
Pocheon	roadside	37.4517, 127.1005	1997-2006	1/10

Seoul	roadside	37.3503, 127.0126	1997-2015	1/19
Ukraine				
Dudarkiv, Boryspil district	abandoned field	50.4507, 30.9642	1996-2011	8/16
Kiev, Novobilychi	roadside	50.4747, 30.3384	1997-2008	4/12
Baryshivka	along railway	49.2186, 28.5265	1996-2005	5/10
Baryshivka	agricultural field	50.2745, 31.3325	2000-2008	5/9
Vinnytsia	roadside	49.2186, 28.5265	1997-2004	6/8

Supplementary Table S2. Designations, place and date of collection and herbarium and NCBI GenBank accession numbers of the *Cryptophyllachora* specimens included in the molecular work.

Sample designa-tion	Place of collection	Geographic coordinates	Date of collection	Herbarium accession number	GenBank accession number ITS/SSU/LSU
Hu-1	Etyek, Hungary	47.4508, 18.7481	21 July 1999	-	MH155433/ MH155451/ MH155469
Hu-2	Esztergom, Hungary	47.7324, 18.7385	15 August 2002	-	MH155434/ MH155452/ MH155470
Hu-3	near Hatvan, Hungary	47.6694, 19.6229	9 September 2003	K(M) 235112	MH155435/ MH155453/ MH155471
Hu-4	Kál, Hungary	47.7200, 20.2825	5 October 2004	-	MH155436/ MH155454/ MH155472
Hu-5	Budapest, Soroksár, Hungary	47.3976, 19.1527	18 August 2005	-	MH155437/ MH155455/ MH155473
Hu-6	Biatorbágy, Hungary	47.4789, 18.8153	8 September 2006	-	MH155438/ MH155456/ MH155474
Hu-7	Biatorbágy, Hungary	47.4789, 18.8153	5 October 2007	-	MH155439/ MH155457/ MH155475

Hu-8	Budaörs, Hungary	47.4604, 18.8947	2 October 2008	BPI 880510	MH155440/ MH155458/ MH155476
Ko-1	Pocheon, Korea	37.4517, 127.1005	2 Sept 2003	SMK19592, BPI 880505, K(M) 235111	MH155441/ MH155459/ MH155477
Ko-2	Seoul, Korea	37.3503, 127.0126	6 Sept 2003	SMK19613, BPI 880506	MH155442/ MH155460/ MH155478
Ko-3	Pyeongchang, Korea	37.5559, 128.4851	22 September 2016	-	MH155443/ MH155461/ MH155479
Ukr-1	Dudarkiv, Boryspil district, Ukraine	50.4507, 30.9642	15 September 2005	BPI 880507, K(M) 235110	MH155444/ MH155462/ MH155480
Ukr-2	Kyiv, Novobilychi, Ukraine	50.4747, 30.3384	25 September 2005	BPI 880508	MH155445/ MH155463/ MH155481
US-1	Trifton, GA, USA	31.477953, -83.440278	19 August 2005	BPI 880509	MH155446/ MH155464/ MH155482
US-2*	Clermont, FL, USA	28.629250, -81.695533	3 June 2014	IMI 505215	MH155447/ MH155465/ MH155483

US-3*	Clermont, FL, USA	28.629250, -81.695533	3 June 2014	IMI 505215	MH155448/ MH155466/ MH155484
US-4*	Clermont, FL, USA	28.629250, -81.695533	3 June 2014	IMI 505215	MH155449/ MH155467/ MH155485
US-5	near Paradise Heights, FL, USA	28.604817, -81.547417	3 June 2014	IMI 505216	MH155450/ MH155468/ MH155486

*Different parts of the infected plant material collected in the same place and at the same time were treated as separate samples during the DNA work to reveal any potential genetic diversity in the pathogen.

Supplementary Table S3. Accession numbers, host plant species, and place and date of collection of herbarium specimens deposited as *Phyllachora ambrosiae* at U.S. National Fungus Collections (BPI) and examined in this work.

Herbarium accession number	Host plant species	Place of collection	Date of collection
BPI 636213	*Ambrosia artemisiifolia* (?)	Colombia	24 July 1940
BPI 636214	*A. artemisiifolia* (?)	Colombia	6 August 1910
BPI 636220	*A. artemisiifolia*	Tuskegee, AL, USA	7 August 1935
BPI 636221	*A. artemisiifolia*	Shenandoah National Park, VA, USA	18 August 1938
BPI 636222	*A. artemisiifolia*	Woods Hole, MA, USA	not specified
BPI 636223	*A. artemisiifolia*	Amery, WI, USA	15 July 1959
BPI 636224	*A. artemisiifolia*	Pine Bluff, WI, USA	9 August 1962
BPI 636225	*A. artemisiifolia*	Gainesville, FL, USA	28 October 1912
BPI 636226	*A. peruviana*	El Cobre, Venezuela	10 September 1932
BPI 636227	*A. peruviana*	Ibaque, Colombia	20 June 1929
BPI 636228	*A. peruviana*	Trujillo, Venezuela	27 August 1932
BPI 636236	*A. tenuifolia*	unknown	16 May 1905
BPI 636240	*A. trifida*	WI, USA	17 September 1905
